# Comparison of [^18^F]DCFPyL and [^18^F]FAPI-74 PET/CT imaging in metastatic prostate cancer

**DOI:** 10.1007/s00259-026-08028-5

**Published:** 2026-06-26

**Authors:** Anniina Hyväkkä, Marko Seppänen, Maria Sundvall, Veera Sivonen, Kalle Mattila, Mikael Anttinen, Otto Ettala, Mikael Högerman, Anna Kirjavainen, Olli Eskola, Sarita Forsback, Virva Saunavaara, Samuli Arvola, Kusti Kilpinen, Pekka Taimen, Simona Malaspina, Heikki Minn

**Affiliations:** 1 Department of Oncology and FICAN West Cancer Center, University of Turku and Turku University Hospital, Turku, Finland; 2 Department of Clinical Physiology, Nuclear Medicine and Turku PET Centre, University of Turku and Turku University Hospital, Turku, Finland; 3 Comprehensive Cancer Center, Helsinki University Hospital, Helsinki, Finland; 4 Department of Urology, University of Turku and Turku University Hospital, Turku, Finland; 5 Radiopharmaceutical Chemistry, Turku PET Centre, University of Turku, Turku, Finland; 6 Department of Medical Physics and Turku PET Centre, Turku University Hospital, Turku, Finland; 7 Department of Medical Physics, Turku University Hospital, Turku, Finland; 8 Institute of Biomedicine, University of Turku, Turku, Finland; 9 Department of Pathology, Tyks Laboratories, Turku University Hospital, Turku, Finland

**Keywords:** Prostate cancer, Prostate-specific membrane antigen (PSMA), ^18^F-DCFPyL, Fibroblast activation protein (FAP), ^18^F-FAPI-74, PET/CT.

## Abstract

**Purpose:**

To compare prostate-specific membrane antigen (PSMA) and fibroblast activation-protein inhibitor (FAPI) targeted PET/CT imaging to assess possible theranostic combination strategies of both tracers in metastatic prostate cancer (mPC).

**Methods:**

This prospective study enrolled patients with mPC who underwent long-axial field-of-view whole-body PET/CT with ^18^F-DCFPyL and ^18^F-FAPI-74 within two weeks. Fifteen patients had hormone sensitive (mHSPC) and 24 castration-resistant (mCRPC) disease. Scans were reviewed according to predefined criteria, with up to five metastases per anatomical region evaluated. Lesions were categorized in four groups as skeletal, pelvic nodal, extrapelvic nodal, or visceral. SUVmax and volumetric parameters were measured for each lesion and at the patient level.

**Results:**

A total of 1365 lesions were identified in 39 patients. ^18^F-DCFPyL PET/CT detected 1351 lesions (99%). In contrast, ^18^F–FAPI-74 identified only 788 (58%) lesions in the PSMA-guided analysis and 523 (38%) lesions in the independent analysis. Median lesion SUVmax was higher for PSMA (24) than for FAPI (5.4) (p < 0.001). Visceral metastases constituted 3% of lesions with both tracers and tended to be more often FAPI-avid (64%) compared to other lesion groups. Only 14 (1%) of lesions were FAPI-positive and PSMA-negative. In whole-tumor burden analysis, median tracer-positive tumor volume on ^18^F-DCFPyL PET/CT was 85 mL and on ^18^F-FAPI-74 PET/CT only 10 mL (p < 0.001).

**Conclusion:**

In mPC, PSMA PET/CT remains the preferred molecular imaging method. Our findings suggest only a limited role of FAPI radioligand therapy. FAPI theranostics should focus on patients with visceral metastases and specific histological subtypes beyond acinar adenocarcinoma.

**Clinical Trial Registration: EudraCT:**

2022–003788-13. Registered 21 September 2023

## Introduction

Prostate cancer (PC) is the second most diagnosed malignancy and the sixth leading cause of cancer-related mortality among men worldwide [1]. Standard treatment for metastatic castration-naïve PC includes androgen deprivation therapy (ADT) combined with androgen receptor signaling inhibitors (ARSI) and the addition of taxane chemotherapy in selected patients [2]. Despite initial responses, most patients eventually progress to metastatic castration-resistant prostate cancer (mCRPC), for which no curative treatments are available.

Prostate-specific membrane antigen (PSMA) is a transmembrane glycoprotein that is markedly overexpressed in malignant prostate epithelial cells and its expression levels correlate with tumor aggressiveness [3]. Owing to its high sensitivity, PSMA has become a clinically validated target for positron emission tomography (PET) imaging to detect PC [4]. Both fluorine-18 and gallium-68 isotopes are used for radiolabeling PSMA-targeting ligands. The superiority of PSMA-PET/CT over conventional imaging, including bone scintigraphy and computed tomography (CT) in the primary staging of men with high-risk PC has been convincingly demonstrated [5]. Furthermore, PSMA-targeted radionuclide therapies have emerged as approved treatments for mCRPC and are promising in earlier phases of disease as well. The phase III VISION trial showed that lutetium-177–labeled PSMA-617 significantly improved both overall survival (15.3 vs 11.3 months) and radiographic progression-free survival compared with standard care alone in mCRPC [6]. However, the success of PSMA-targeting therapeutic approaches is limited by treatment resistance. This is in part due to heterogeneous PSMA expression and the fact that approximately 10–20% of metastatic lesions may lack PSMA expression entirely [7].

The tumor microenvironment (TME) plays a critical role in regulating cancer behavior and is increasingly recognized as a potential therapeutic target. Cancer-associated fibroblasts (CAFs) represent one of the most abundant cellular components of TME and influence key oncogenic processes, including angiogenesis, invasion, metastatic spread, and therapeutic resistance [8, 9]. Fibroblast activation protein (FAP) is an atypical type II transmembrane serine protease highly expressed on CAFs across most cancer types and has emerged as a promising molecular target for both imaging and therapy. Accordingly, PET/CT imaging using various fibroblast activation protein inhibitor (FAPI) compounds has demonstrated promise to detect several solid malignancies [10, 11]. In line with PSMA, FAPI offers a potential theranostic option labeled with beta- and alpha-emitter radionuclides [12, 13].

FAP expressions have also been shown in PC although large patient cohorts have not been systematically evaluated with radionuclide imaging [14–16]. The ability of FAPI-PET/CT to detect malignant lesions independent of tumor cell PSMA expression highlights its potential complementary role in PC. This would be valuable especially in patients who show primary or secondary resistance to PSMA-radioligand therapy (RLT) given that metastatic presentation may be very heterogenous at molecular and functional level after development of mCRPC [7]. Preliminary studies have suggested a role for FAPI RLT in mCRPC [13]. We therefore evaluated the potential utility of ^18^F-labeled FAPI-74 for detecting metastatic lesions in patients with PC and undertook a comparison of uptake of ^18^F-FAPI PET/CT to ^18^F-DCFPyL (PSMA) PET/CT. Our prospective study was focused on future theranostic applications where both PSMA and FAPI emerge as potential targets in patients progressing after standard oncologic therapy.

## Patients and methods

### Study design

This investigator initiated prospective, single-institutional trial (EudraCT Number: 2022–003788-13, 2023–09–21) compared PSMA-PET/CT (^18^F-DCFPyL) and FAPI-PET/CT (^18^F-FAPI-74) in PC. Study included 40 patients, 15 men with metastatic hormone sensitive PC (mHSPC) and 25 men with mCRPC. Patients underwent PET/CT twice within 2 weeks in random order with ^18^F-DCFPyL or ^18^F-FAPI-74 at the University Hospital of Turku, PET Centre between January 2024 and August 2025. The study was approved by the National Committee on Medical Research Ethics (Tukija) on March 7, 2023, by Finnish Medicines Agency (FIMEA) on Sept 21, 2023, and by Wellbeing Services County of Southwest Finland (VARHA) on January 24, 2024. All patients signed the Ethical Board approved informed consent and data was handled according to principles in compliance with General Data Protection Regulation (GDPR).

Inclusion criteria were 50–85 years old men with histologically confirmed PC and WHO performance score 0–2. They were required to present with at least one distant metastasis detected on CT, magnetic resonance imaging (MRI), bone scintigraphy or single-photon emission computed tomography (SPECT). All previous treatment lines were allowed, but at least one week’s wash out from previous treatment (except ADT) was mandated and any change in the treatment of PC was prohibited between the scans. Exclusion criteria included inability to understand the purpose of the study and medical conditions precluding whole-body PET/CT imaging.

Patients’ clinical characteristics such as age, dates for primary diagnosis of PC and its metastases, Gleason score, primary prostate-specific antigen (PSA) and alkaline phosphatase (ALP), type(s) of metastases (bone/node/visceral) and previous therapies were collected. For each patient, Charlson Comorbidity Index (CCI) was defined at the time of study enrollment. Study data were collected and managed with the REDCap secure electronic data capture tool (project-redcap.org).

### Radiopharmaceuticals and PET/CT

Fluorine-18 labeled PSMA (^18^F-DCFPyL) was purchased from Curium Finland Oy, (www.curiumfinland.fi). The median activity of intravenously injected ^18^F-DCFPyL was 329 MBq (IQR 323–334). FAP-targeting tracer ^18^F-FAPI-74 was synthesized on-site at Turku PET Centre using Trasis AllInOne platform and single-use synthesis cassettes (Ans, Belgium). FAPI-74 precursor and reference compound were obtained from SOFIE Biosciences (Dulles, VA, USA). The median activity of intravenously injected ^18^F-FAPI-74 was 250 MBq (IQR 244–257). Patients were instructed to rest prior to imaging. Fasting was not required. PET/CT imaging was performed using a long axial field-of-view system (Siemens Biograph Vision Quadra, Knoxville, TN, USA). PET acquisitions started 60 min after injection and consisted of a 4-min static single-bed-position acquisition covering the area from the skull to the mid-thighs, with patients imaged supine and arms raised. Images were reconstructed using UltraHD PET in high-sensitivity mode (4 iterations, 5 subsets), with an all-pass filter for FAPI and a 2-mm Gaussian filter for DCFPyL. Data were corrected for dead time, randoms, scatter, decay, and attenuation. Patients’ vital signs were monitored during imaging and 15 min thereafter.

### Image evaluation

The PET/CT imaging findings were evaluated separately and in a blinded manner by two experienced nuclear medicine physicians (M.Se., S.M), each reader interpreting one tracer modality according to predefined criteria. Visual assessment was followed by measurement of maximum standardized uptake values (SUVmax) in a volume of interest adjusted to the size of metastasis. Metastatic lesions were categorized into four anatomical groups: skeletal, pelvic lymph nodes, extra-pelvic lymph nodes, and visceral metastases.

Lesion positivity for metastases was defined using tracer-specific uptake criteria. On ^18^F-DCFPyL PET/CT, skeletal, liver, mediastinal and subclavicular nodal metastases were considered positive if SUVmax exceeded liver activity, whereas pelvic and retroperitoneal nodal metastases as well as all visceral lesions (except for liver) were considered positive if SUVmax exceeded blood-pool activity [17, 18]. On ^18^F-FAPI-74 PET/CT, lesion positivity for all metastatic categories was defined as SUVmax greater than blood-pool activity in sites incompatible with physiological distribution or known or suspected site of inflammatory change. Up to five of the most avid metastases per anatomical region were measured. The anatomical regions included the skull; the cervical, thoracic, and lumbar spine; sternum, clavicle, scapula, ribs, humerus, sacrum, ilium, ischium, and femur; lymph node regions (common, external, and internal iliac; obturator, presacral, and perirectal; retroperitoneal, mediastinal, and supraclavicular); and visceral organs including the liver, lungs, and adrenal glands.

Subsequently, a separate lesion-level comparative paired analysis was performed to assess the correspondence between ^18^F-DCFPyL and ^18^F-FAPI-74 uptake. SUVmax was first measured from PSMA-positive lesions, after which the corresponding anatomical location was identified on the co-registered FAPI PET/CT images and SUVmax was measured using an identical region of interest. In addition, a complementary ^18^F-FAPI-74-guided analysis was performed to assess ^18^F-DCFPyL uptake in metastatic lesions identified only on ^18^F-FAPI-74 PET and with an anatomical correlate, thereby enabling the identification of FAPI-positive but PSMA-negative lesions.

Finally, whole-tumor burden analysis was performed for both tracers. For PSMA lesion positivity was defined using an SUV threshold of 5.8 (above cohort’s median liver activity). A higher threshold of 11.6 (twice the cohort’s median liver uptake) was applied for liver metastases. For FAPI, lesion positivity was defined using an SUV threshold of 4.1, corresponding to 1.5-fold uptake above blood-pool activity. Image analysis was performed using Hermia Molecular Imaging Software, Affinity 4.0 (Hermes Medical Solutions, Stockholm, Sweden).

### Histology and immunohistochemical analysis

Biopsy specimens from a lung metastasis of a patient with mHSPC were fixed in formalin, embedded in paraffin, and sectioned at 4 µm thickness for routine haematoxylin and eosin (H&E) staining and immunohistochemistry. Antigen retrieval was carried out by microwaving the slides (7 min 600 W + 7 min 450 W + cool down 20 min) in Tris–EDTA HIER solution, pH 9.0 (TES999, ScyTek Laboratories, Logan, UT, USA). After endogenous peroxidase block in 3% hydrogen peroxide for 10 min in room temperature (RT) and protein blocking with Normal antibody diluent (BD09-125, WellMed, Arnhem, Netherlands) for 10 min in RT, rabbit monoclonal anti-PSMA antibody (1:200, clone D7I8E, Cell Signaling technology, Leiden, Netherlands) and rabbit monoclonal anti-FAP antibody (1:250, clone EPR20021, Abcam, Cambridge, UK) were used as primary antibodies for 1 h at RT. BrightVision 1-step detection system goat-anti-rabbit HRP (DPVR110HRP, WellMed) and BrightDAB (BS04-110, WellMed) were used for detection of the primary antibodies for 30 min and 10 min at RT, respectively. The sections were counter stained with Meyer's hematoxylin for 1 min at RT and mounted in Pertex. Both immunostainings were carried out with Lab Vision autostainer (Thermo Fisher Scientific, Waltham, MA, USA). PSMA-positive primary PC and invasive bladder cancer tissue were used as positive controls for PSMA and FAP stainings, respectively.

### Statistical analysis

Clinical variables (age, CCI, PSA, ALP, Gleason and prior treatments), imaging parameters (Liver- and Blood -SUVmax, SUVmean, SUVpeak and tumor volume) and uptake of radiotracers ^18^F-DCFPyL and ^18^F-FAPI-74 PET/CT) across different anatomical groups (skeletal, pelvic nodal, extrapelvic nodal, and visceral) were summarized with descriptive statistics. SUVmax and the number of detected metastatic lesions were visualized using box plots and bar plots, respectively, grouped by radiotracer and anatomical groups. Generalized linear mixed models were used to analyse the associations between imaging parameters and radiotracers. For continuous SUVmax values, an inverse Gaussian distribution with a log link was applied, while a binomial distribution was used to study association between the detection status of metastatic lesions and clinical state (mHSPC vs. mCRPC). All models accounted for repeated measurements by including subjects and anatomical groups as random effects.

The normality of variables was evaluated visually and tested with the Shapiro–Wilk test. Due to the non-normality of the continuous variables, nonparametric methods were used. The statistical significance level was set at 0.05 in all tests (two-tailed) and 95% confidence intervals (CI) were calculated. The analyses were performed using RStudio, version 2024.04.2 based on R, version 4.4.1.

## Results

### Patients

Forty patients were enrolled, of which 39 (15 with mHSPC and 24 with mCRPC) successfully completed the study protocol. The only protocol failure occurred to a man with mCRPC, where the radiopharmaceutical quality did not meet the demands set for ^18^F-FAPI-74. The median age of patients was 73 years (range 61–83), and their clinical characteristics are summarized in Table 1. All patients with mHSPC had initiated ADT a median of 22 days (IQR 15–32) before imaging but none had received chemotherapy, ARSI or RLT. In contrast, all patients with mCRPC had undergone one or two lines of taxane chemotherapy, with some also received one or two lines of ARSI and three patients had undergone ^177^Lu-PSMA-617 RLT. The median interval between the two PET/CT imaging studies was 9 days (range 3–17). All patients tolerated the imaging well and none of the patients reported subjective symptoms during or immediately after studies.

**Table 1:** Patient characteristics

	All patients n = 39	mHSPC n = 15	mCRPC n = 24
Age (years)^a^	73 (61–83)	73 (61–82)	75 (63–83)
Charlson Comorbidity Index (CCI)^a^	6 (6–12)	6 (6–8)	8 (6–12)
PSA (ng/ml)^a^	55 (1–2 000)	55 (3–2 000)	47 (1–280)
ALP (U/l)^a^	106 (35–1 961)	199 (76–1 961)	94 (35–510)
Gleason score (n, %)			
< 9	10 (26%)	4 (27%)	6 (26%)
4 + 5	19 (50%)	7 (47%)	12 (52%)
5 + 4	9 (24%)	4 (27%)	5 (22%)
unknown	1	0	1
Treatments before imaging (n, %)			
Prostatectomy	8 (21%)	1 (7%)	7 (29%)
Radical radiotherapy	14 (36%)	3 (20%)	11 (46%)
Docetaxel	24 (62%)	0	24 (100%)
Cabazitaxel	7 (18%)	0	7 (29%)
Abiraterone	8 (21%)	0	8 (33%)
Enzalutamide	16 (41%)	0	16 (67%)
^177^Lu-PSMA-617	3 (8%)	0	3 (13%)

^a^ Median (range)

PSA = prostate-specific antigen, ALP = alkaline phosphatase

### Independent analysis of PSMA and FAPI PET/CT images

A total of 1351 PSMA-positive and 523 FAPI-positive lesions in 39 men were identified with ^18^F-DCFPyL PET/CT and with ^18^F-FAPI-74 PET/CT according to predefined protocol criteria. The majority of detected metastases were skeletal (PSMA 77%, n = 1045; FAPI 78%, n = 407), while pelvic and extrapelvic nodal metastases represented 10% (n = 136; n = 149, respectively), of the lesions on PSMA imaging. In contrast, among lesions detected on FAPI PET/CT, only 7% (n = 35) were pelvic and 12% (n = 65) extrapelvic nodal metastases. In other words, FAPI PET/CT tended to show a higher proportion of extrapelvic relative to pelvic nodal metastases compared with PSMA PET/CT. Visceral metastases constituted 3% of all detected lesions with both tracers (PSMA: n = 38; FAPI: n = 16).

**Fig. 1:**
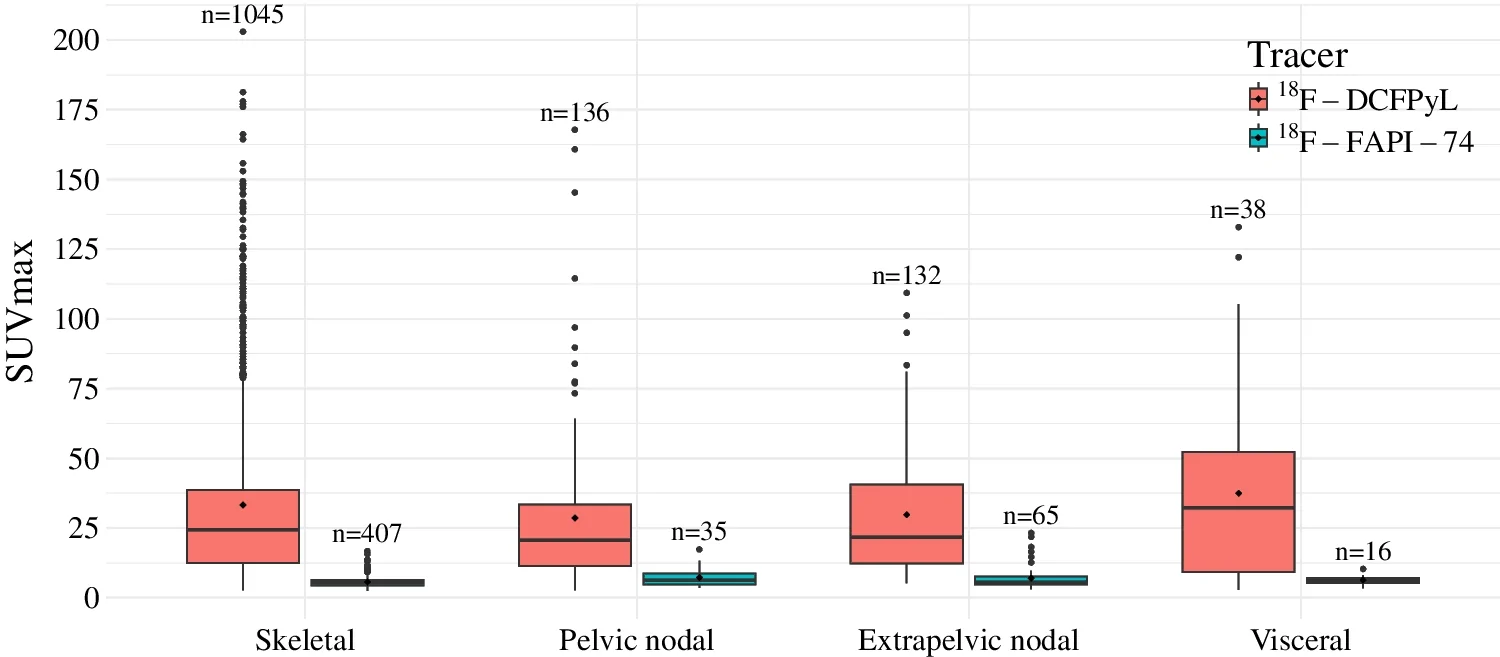
Box plots demonstrating SUVmax uptake of ^1^⁸F-DCFPyL and ^1^⁸F-FAPI-74 in prostate cancer (PC) metastatic lesions across different anatomical groups. Overall, ^1^⁸F-DCFPyL demonstrated significantly higher uptake compared with ^1^⁸F-FAPI-74, while no statistically significant differences were observed within the same tracer across anatomical groups (boxes indicate IQR with median; whiskers indicate range; numbers detected lesion counts)

SUVmax of metastatic lesions was measured, and, overall, lesions demonstrated significantly higher uptake on ^18^F-DCFPyL compared with ^18^F-FAPI-74 (SUVmax median 24 vs. 5.4; p < 0.001) (Fig. 1). Neither tracer showed differential uptake in sclerotic versus lytic bone lesions. The differences in median SUVmax values among individual metastatic groups are given in Table 2, which also summarizes the corresponding lesion tumor-to-background uptake ratios.

**Table 2:** Comparison of ^18^F-DCFPyL and ^18^F-FAPI-74 uptake in prostate cancer. SUVmax values of metastatic lesions derived from independent analysis of PSMA and FAPI PET/CT images

	n	^18^F-DCFPyL	n	^18^F-FAPI-74
Liver-SUVmax^a^	39	5.6 (4.3–6.7)	39	1.4 (1.3–1.8)
Blood pool-SUVmax^a^	39	1.7 (1.4–2.0)	39	2.6 (2.2–3.4)
Lesion-SUVmax^a^				
Overall	1351	24 (12–39)	523	5.4 (4.5–6.6)
Skeletal	1045	24 (12–39)	407	5.3 (4.4–6.3)
Pelvic nodal	136	21 (11–34)	35	6.3 (4.8–8.8)
Extrapelvic nodal	132	22 (12–41)	65	5.5 (4.7–7.6)
Visceral	38	32 (9–53)	16	6.3 (5.2–7.2)
Lesion Tumor-to-Background uptake ratio^a,b^				
Skeletal	1045	5 (3–9)	407	1.8 (1.5–2.4)
Pelvic nodal	136	13 (7–28)	35	2.8 (2.0–3.2)
Extrapelvic nodal Mediastinal nodes Retroperitoneal nodes Subclavicular nodes	132 32 71 29	9 (5–19) 6 (4–10) 15 (8–26) 3 (2–5)	65 15 40 10	2.2 (1.6–2.9) 3.6 (2.5–4.2) 2.2 (1.6–2.7) 1.5 (1.2–2.1)
Visceral Liver Lungs	38 14 24	6 (3–47) 5 (2–47) 8 (3–40)	16 3 13	2.4 (2.0–3.1) 1.8 (1.8–2.5) 2.7 (2.4–3.1)

^a^Median (IQR)

^b^On ^18^F-DCFPyL PET/CT, skeletal, mediastinal nodal, subclavicular nodal, and liver metastases are reported as lesion-to-liver SUVmax ratios; all other metastatic sites are reported as lesion-to-blood-pool SUVmax uptake ratios. On ^18^F-FAPI-74 PET/CT, all values are reported as lesion-to-blood-pool SUVmax uptake ratios

SUV = Standardized uptake value

### Paired analysis of PSMA and FAPI PET/CT studies

**Fig. 2:**
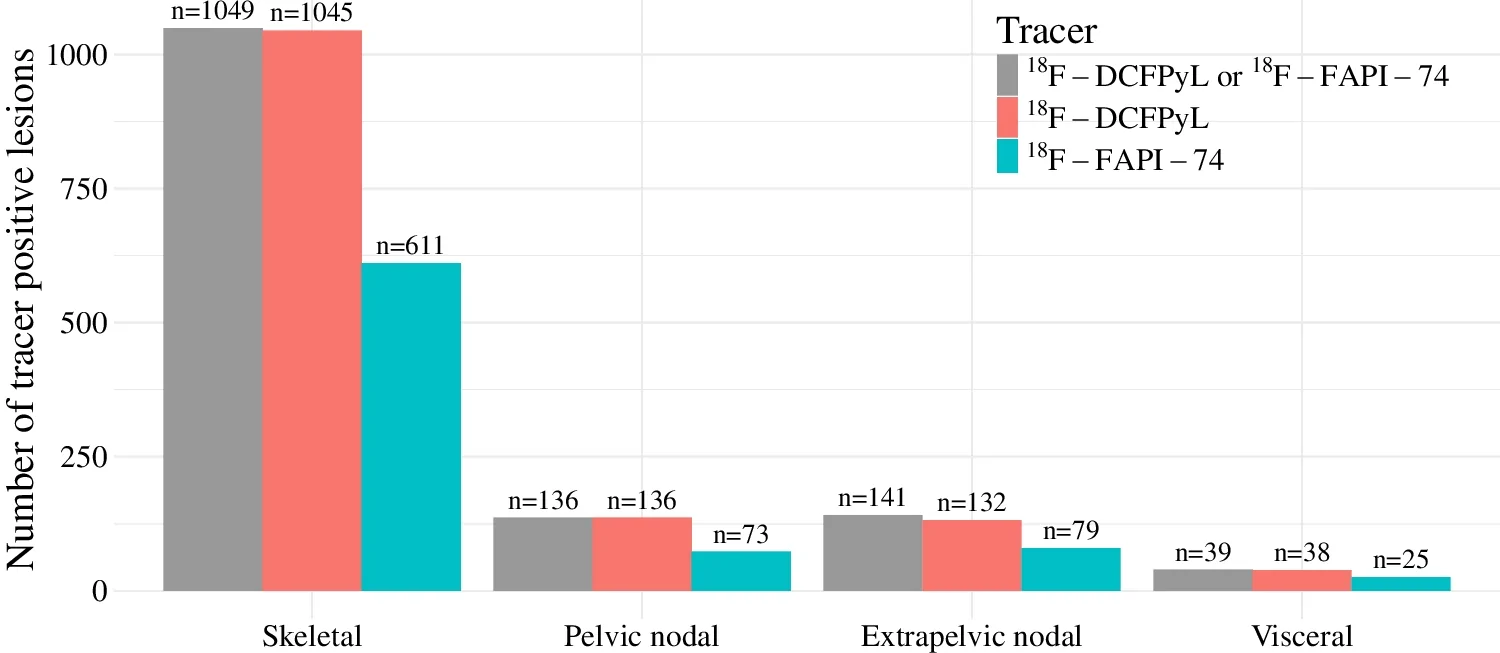
Bar charts illustrating the number of tracer-positive metastatic lesions detected with ^18^F-DCFPyL and ^18^F-FAPI-74- across different anatomical groups (skeletal, pelvic nodal, extrapelvic nodal, and visceral) in the paired PET/CT analysis where PSMA findings were available for reading of ^18^F-FAPI-74 PET/CT

When lesions detected on both PET/CT modalities were combined, a total of 1365 unique lesions positive on either ^18^F-DCFPyL- or ^18^F-FAPI-74-positive) were identified (Fig. 2). ^18^F-DCFPyL imaging detected 99% (n = 1351) of lesions with no difference between mHSPC (n = 657) and mCRPC (n = 694) (p = 0.976). In contrast, ^18^F-FAPI-74 positive lesions constituted less than two thirds (58%; n = 788) of all detected metastases and similar to PSMA imaging, there was no significant difference between mHSPC (63%; n = 418) and mCRPC (53%; n = 370) (p = 0.322). Note that without PSMA guidance many lesions would have remained undetected or considered as equivocal while independent reading of ^18^F-FAPI-74 PET/CT detected only 523 out of all 1365 tracer positive lesions (38%).

Overall, only 14 (1%) of ^18^F-FAPI-74 positive metastases were negative for ^18^F-DCFPyL. Of these, four (0.4%, 4/1049) were skeletal metastases, nine (6%, 9/141) extra-pelvic nodal and one (3%, 1/39) visceral, respectively. No FAPI-positive and PSMA negative pelvic nodal metastases were observed.

Interestingly, ^18^F-FAPI-74 detected a relatively high proportion of visceral metastases in patients with mHSPC (83%, n = 15/18) compared to the other lesion groups. However, detection rates did not differ significantly between mHSPC and mCRPC (83% vs. 48%, p = 0.397). A more detailed distribution of detected FAPI-positive and PSMA negative lesions between groups is shown in Table 3.

**Table 3:** Paired lesion-level analysis comparing metastatic lesions detected with ^1^⁸F-DCFPyL and ^1^⁸F-FAPI-74 PET/CT

	Skeletal n = 1049		Pelvic n = 136		Extrapelvic n = 141		Visceral n = 39		Overall n = 1365	
	mHSPC n = 514	mCRPC n = 535	mHSPC n = 73	mCRPC n = 63	mHSPC n = 58	mCRPC n = 83	mHSPC n = 18	mCRPC n = 21	mHSPC n = 663	mCRPC n = 702
PSMA pos./FAPI neg	186 (36%)	252 (47%)	31 (42%)	32 (51%)	25 (43%)	37 (45%)	3 (17%)	11 (52%)	245 (37%)	332 (47%)
FAPI pos./PSMA neg	2 (0.4%)	2 (0.4%)	0 (0%)	0 (0%)	4 (7%)	5 (6%)	0 (0%)	1 (5%)	6 (1%)	8 (1%)

### Whole-tumor metastatic burden

**Fig. 3:**
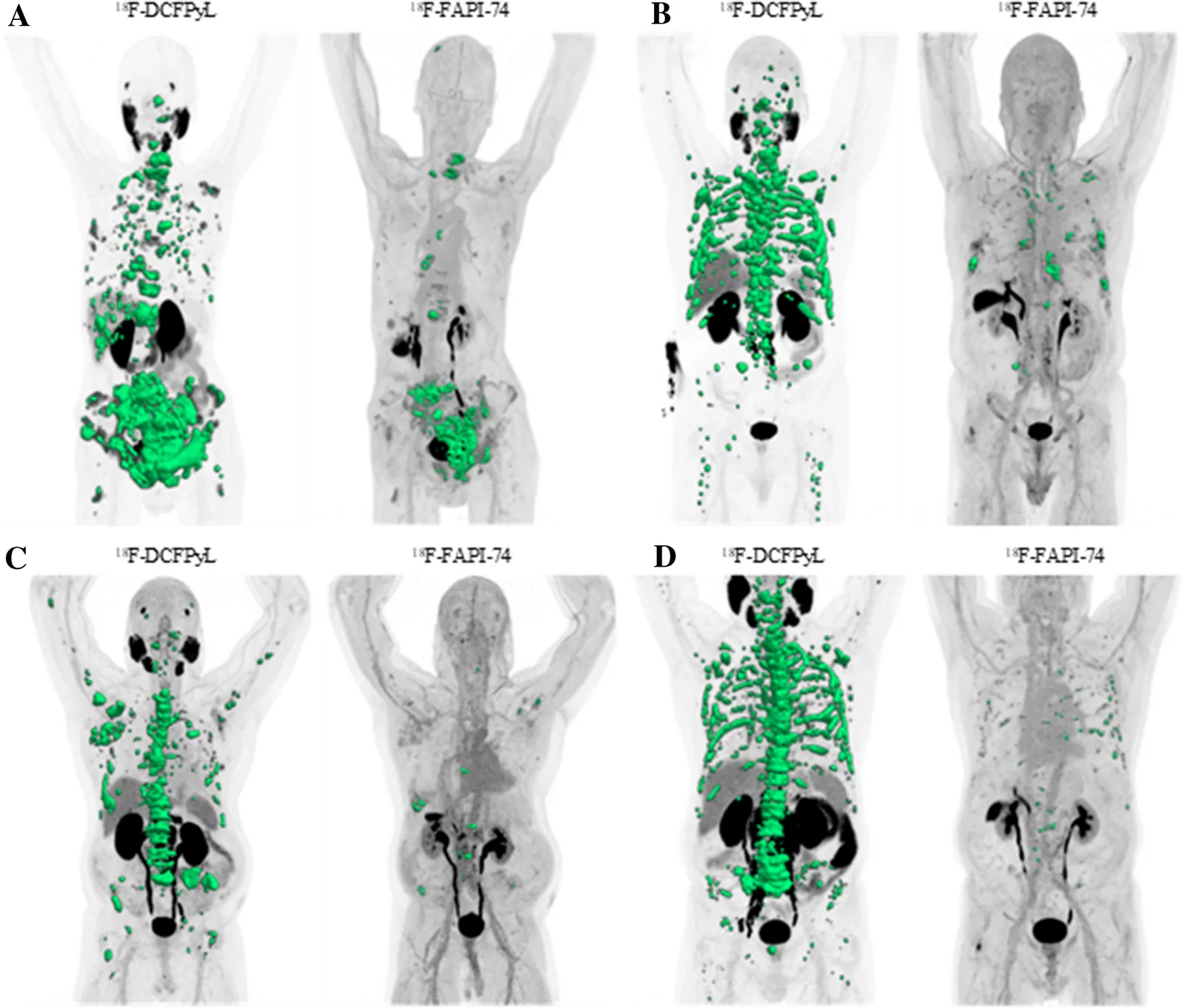
Four representative examples of total tumor volume analyses using ^1^⁸F-DCFPyL and ^1^⁸F-FAPI-74 PET/CT. Panels A and B show patients with hormone sensitive prostate cancer (mHSPC), whereas panels C and D show patients with castration-resistant prostate cancer (mCRPC). Total tumor volume was markedly greater on ^1^⁸F-DCFPyL PET/CT compared with ^1^⁸F-FAPI-74 PET/CT and especially skeletal disease is much more conspicuous on the former tracer

Whole-tumor burden analyses were consistent with the lesion-level findings and showed a higher tumor burden on ^1^⁸F-DCFPyL compared with ^18^F-FAPI-74. This was observed both for patients with mHSPC and mCRPC as illustrated in Fig. 3.

The median tracer-positive whole body tumor volume was almost an order of magnitude higher on ^18^F-DCFPyL PET/CT (85 mL; IQR 26–336 mL) than that on ^18^F-FAPI-74 PET/CT (10 mL; IQR 0–35 mL; p < 0.001). Consistent with this, total lesion uptake expressed as product of volumetric size and SUVmean was greater on ^18^F-DCFPyL PET/CT, with a median value of 1 050 mL (IQR 288–5264 mL), compared with 47 mL (IQR 0–192 mL) on ^18^F-FAPI-74 PET/CT (p < 0.001).

According to the predefined whole-tumor burden criteria, a single patient with mHSPC had no clear detectable ^18^F-DCFPyL uptake, whereas ten patients (6 mCRPC, 4 mHSPC) had no detectable ^18^F-FAPI-74 uptake on whole-tumor burden analysis. Table 4 summarizes the comparison of total tumor volume-based uptake and uptake intensity parameters SUVmax, SUVmean, and SUVpeak. It highlights the generally remarkable difference between uptake of ^18^F-DCFPyL and ^18^F-FAPI-74 in PC metastases which is reflected similarly in individual metastatic groups shown in Fig. 1 and 2.

**Table 4:** Comparison of ^18^F-DCFPyL and ^18^F-FAPI-74 total tumor volume uptake in PC

	^18^F-DCFPyL	^18^F-FAPI-74	p-value^b^
Tumor volume (mL)^a^	85 (26–336)	10 (0–35)	< 0.001
SUVmax^a^	54 (26–79)	8 (0–11)	< 0.001
SUVmean^a^	12 (9–16)	5 (0–5)	< 0.001
SUVpeak^a^	27 (15–43)	5 (0–7)	< 0.001
Total tumor volume (mL)^a^	1 050 (288–5 264)	47 (0–192)	< 0.001

^a^Median (IQR)

^b^Paired Wilcoxon signed rank test

SUV = Standardized uptake value

### Histology and immunohistochemistry

**Fig. 4:**
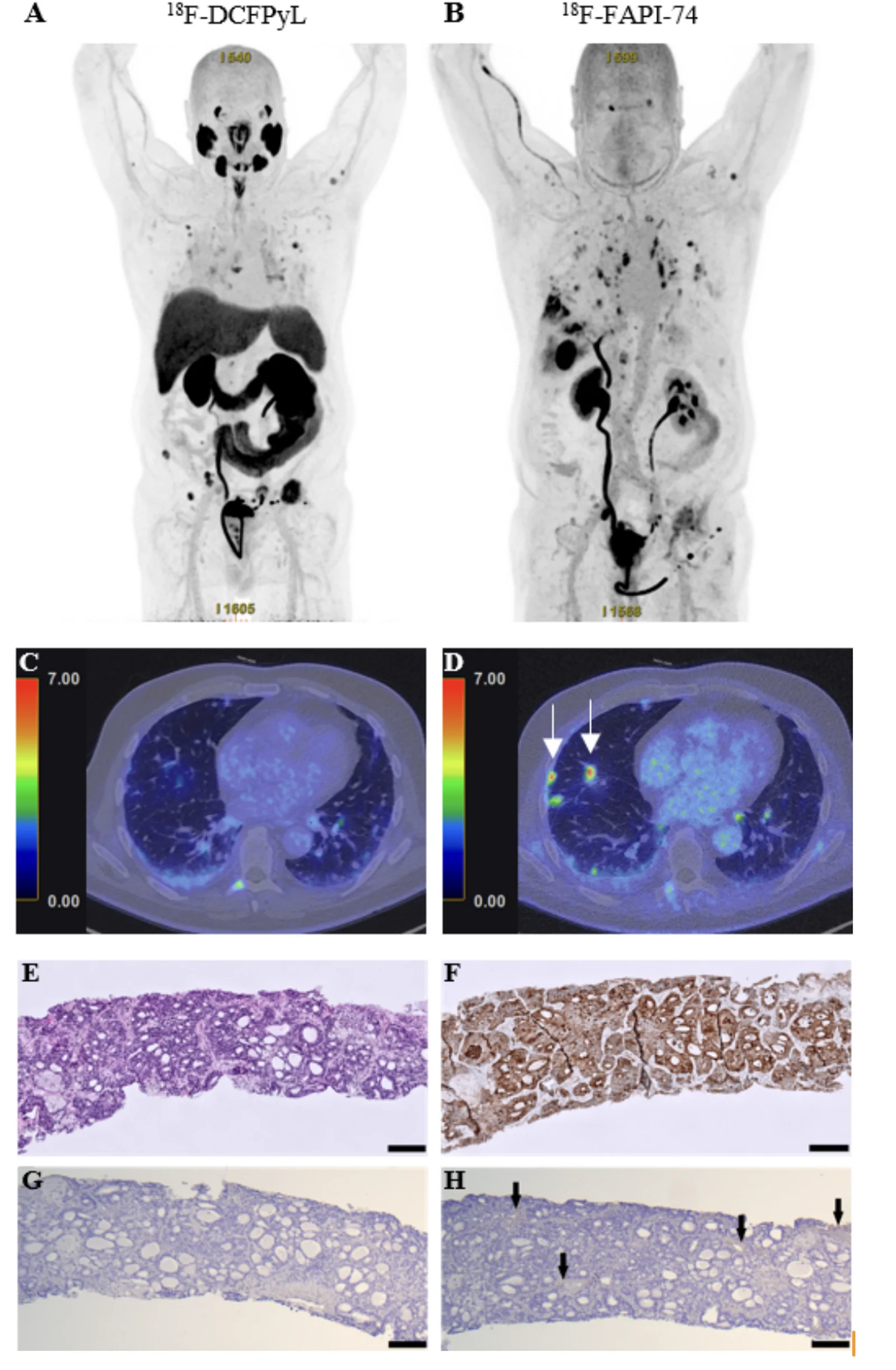
Maximum-intensity ^18^F-DCFPyL (A) and ^18^F-FAPI-74 (B) PET/CT projection images of a 73-year-old man with mHSPC. The corresponding thoracic axial views show two ^18^F-DCFPyL-negative lung metastases (C) which have uptake on ^18^F -FAPI-74 PET/CT (D), indicated with white arrows. The immunohistochemical (IHC) analysis was obtained from biopsy of the lung metastasis showing routine staining with H&E (E) and IHC with antibodies against PSA, PSMA and FAP (F-H). PSA staining is strongly positive in all cancer cells (F). PSMA staining is negative (G) while black arrows indicate stromal areas with very faint FAP staining (H). Scale bars 200 µm.

The protocol included IHC analysis of biopsies from metastases which will be published in more detail in a separate communication. To illustrate PSMA and FAP expressions at the cellular level, we present here a patient with mHSPC and lung metastases (Fig. 4). Although the metastatic PSMA-negative lung nodules demonstrated uptake of ^18^F-FAPI-74, IHC analysis of the tumor biopsy showed only weak positive FAP expression in CAFs within stromal islets surrounding the tumor cells.

## Discussion

This study investigated the clinical utility of ^18^F-labeled FAPI-74 PET/CT for the detection of metastatic lesions in patients with PC. We used ^18^F-DCFPyL as the comparator while specifically foreseeing on theranostic applications of both FAPI and PSMA. Previous studies and case reports involving small patient cohorts have shown the expression of FAP in PC and its metastases using ^68^ Ga-labeled FAPI-04 or FAPI-46 [14–16, 19, 20]. To our knowledge, the present study represents the first prospective evaluation directly comparing PSMA-based PET imaging with FAPI PET imaging using fluorine-18 in the largest cohort of patients with PC across different phases of metastatic progression reported to date.

We found that ^18^F-FAPI-74 is well tolerated and capable of detecting metastatic lesions in both mHSPC and mCRPC, although the diagnostic potential of FAPI-PET/CT turned out to be limited due to the generally much lower metastatic uptake compared to ^18^F-DCFPyL. The inferior detection sensitivity of ^18^F-FAPI-74 was similar in patients with mHSPC and mCRPC. The overall median lesion SUVmax 5.4 of ^18^F-FAPI-74 is in line with prior FAPI PET/CT studies [14, 19, 20]. Although both skeletal and soft-tissue metastases demonstrated uptake of ^18^F-FAPI-74, Fig. 2 and especially Fig. 3 highlight the notable differences between the metastatic presentation of PC on ^18^F-FAPI-74 and ^18^F-DCFPyL PET/CT where the former clearly failed to show the full extent of skeletal disease. In the independent analyses of both tracers, ^18^F-FAPI-74 detected only 39% of metastases compared to those found with ^18^F-DCFPyL.

In the paired analysis, where the detection of metastatic lesions by ^18^F-FAPI-74 PET/CT was supported by concurrent reading of PSMA scans, no statistically significant differences were observed in the detection of skeletal, nodal or visceral metastases between mHSPC and mCRPC, although detection rates for visceral metastases were numerically higher in mHSPC (83%) than in mCRPC (48%). Among the small subset (1%) of PSMA-negative and FAPI-positive lesions, extrapelvic nodal metastases were overrepresented although this may be coincidental due to the limited number of lesions. Overall, 9 of all 141 extrapelvic nodal metastases (6%) were FAPI-positive and PSMA-negative. Our findings do not thus support higher FAPI uptake at later stages of disease although this should be confirmed in sequential imaging of same patients. Our patients with mHSPC had not received systemic treatment other than ADT but presented a comparable FAPI-avid tumor burden to patients with mCRPC (Fig. 3).

PSMA-targeted tracers bind directly to PC cells whereas FAPI tracers generally target FAP expressed on CAFs within the tumor stroma, thereby reflecting stromal activation rather than tumor cell–specific uptake [3, 21]. Previous studies indicate that tissue expression of FAP in PC increases in advanced disease and progression to castration resistant stage has been associated with elevated FAP expression [14]. Huang et al. [22] studied PSMA and FAP expression using IHC in 116 mCRPC tumors. In their analysis the median FAP immunoscore was significantly lower than that of PSMA and FAP expression did not correlate with PSMA expression levels or with PC histologic subtypes. The general observation among solid cancers indicates that FAP expression reflects stromal activity within the TME. Although FAP expressions have been reported in tumor cells in certain malignancies, such as hepatocellular carcinoma and cholangiocarcinoma, expression in PC cells appears to be rare [21, 22]. Similarly, our IHC analysis of a lung metastasis biopsy from a patient with HSPC revealed only minimal FAP expression limited to stromal areas, with no detectable expression in the tumor cells, consistent with the predominantly stromal localization of FAP reported in PC.

Neuroendocrine differentiation of PC has been associated with reduced PSMA expression and, conversely, increased FAP expression in prior studies [23]. Previous FAPI PET/CT studies in PC have included neuroendocrine-transformed subtypes [14, 20] and have reported higher SUVmax in neuroendocrine-differentiated tumors [20]. Again, at IHC of mCRPC no differences were observed in FAP expression across different histologic subtypes [22]. In our cohort, one patient had de novo neuroendocrine-differentiated PC and demonstrated a higher total FAP-avid tumor volume (680 mL vs 47 mL) and a relatively higher SUVmax (15.5 vs 7.8) compared with the cohort median values among mHSPC. Although based on a single case, this observation is in line with previous study [19] and supports the hypothesis of increased relevance of FAPI PET/CT and possibly theranostic application in neuroendocrine-transformed PC. Compared to somatostatin receptor imaging followed by RLT using octreotide analogues FAPI RLT may offer better biodistribution owing to higher metastatic uptake in these patients [24].

This study has several limitations. First, the interpretation of FAPI PET/CT was based on predefined criteria agreed upon by two experienced nuclear medicine physicians. While this approach ensured consistency, these criteria are not yet standardized for PC, which may limit comparability with other studies. Second, in the paired analysis, tracer uptake was quantified for all PSMA-positive lesions, including those with a low FAPI signal. To balance this, only uptake exceeding blood pool background activity was considered positive for the final analysis, reducing the risk of overestimating the number of FAPI-positive lesions. This also highlights that if the analysis was based solely on visual assessment of FAPI PET images, some lesions with low-level uptake might remain undetected. Third, FAP is also overexpressed in several non-oncological conditions, such as chronic inflammation and arthritis [25, 26]. Consequently, FAPI tracers may therefore bind to benign processes, complicating the differentiation between benign and malignant FAPI-only–positive lesions. To minimize false FAPI-only-positive findings, FAPI uptake was quantified only in PSMA-negative lesions with a corresponding anatomical correlate consistent with metastasis. Fourth, whole-tumor burden calculations were based on predefined SUV thresholds, as no prior established consensus criteria for FAPI exist, therefore thresholds were defined for the purpose of current study. Indeed, unlike in lung and urothelial carcinoma, there are no universally accepted criteria defining FAPI-positive lesions in PC, highlighting the need for disease-specific standardization [27] Finally, histopathological confirmation was generally unavailable for metastatic lesions. In the next phase of the study, we aim to systemically analyze those cases in which biopsy confirmation from metastasis is available.

## Conclusion

We conclude that the role of FAPI theranostics in PC appears to be generally limited due to generally much lower tracer uptake than that of PSMA especially in bone and nodal metastases which mostly lack the stromal component in TME. However, prospective multicenter studies of patient populations presenting with PSMA-negative visceral metastases and neuroendocrine-differentiated PC are warranted taking into account the differences of FAPI-ligands in their theranostic potential [28]. While our findings highlight the dominant role of PSMA-based imaging and theranostics in metastatic PC, FAPI PET/CT may possibly provide complementary value in biologically distinct subgroups showing FAPI-avid but PSMA-negative metastases.

## Data Availability

The datasets generated during and/or analysed during the current study are available from the corresponding author on reasonable request.

## Abbreviations

ADT: Androgen deprivation therapy
ARSI: Androgen receptor signal inhibitor
ALP: Alkaline phosphatase
CAFs: Cancer-associated fibroblasts
CCI: Charlson comorbidity index
CT: Computed tomography
FAP: Fibroblast activation protein
FAPI: Fibroblast activation protein inhibitor
IHC: Immunohistochemistry
IQR: Interquartile range
MRI: Magnetic resonance imaging
PC: Prostate cancer
mHSPC: Metastatic hormone sensitive prostate cancer
mCRPC: Metastatic castration resistant prostate cancer
PET: Positron emission tomography
PSA: Prostate-specific antigen
PSMA: Prostate-specific membrane antigen
RLT: Radioligand therapy
RT: Room temperature
SPECT: Single photon emission computed tomography
SUV: Standardized uptake value
TME: Tumor microenvironment
